# Puerperal Eclampsia; High Forceps Operation; Recovery

**Published:** 1882-03

**Authors:** Theodore Trumbull

**Affiliations:** Monticello, Florida


### Original Communications.

---

ARTICLE II.

### PUERPERAL ECLAMPSIA; HIGH FORCEPS OPERATION; RECOVERY. By THEODORE TRUMBULL, M.D., of Monticello, Florida.

Carrie B., æt. seventeen, primipara, was delivered on October 20, 1881. Her labor commenced by the discharge of the liquor amnii, about one o'clock on Monday morning. On the Tuesday prior to her confinement she complained of severe cephalalgia, dim vision, and transient dizziness, spots before the eyes and œdema of the subcutaneous cellular tissue, especially of the face. On the morning of her delivery she awakened with complete loss of sight, and soon thereafter was seized with a convulsion. She had eight strong convulsions between six and twelve A. M., at which time I arrived. I gave chloroform inhalations at short intervals; not keeping her completely anæsthetized. She had bitten her tongue, and was somewhat semi-stupefied ere the administration of the chloroform. She was again seized at 12:30, the attack lasting one minute and fifteen seconds, with frothy saliva collecting about her mouth, pulse full and carotids bounding; the whole appearance was so changed as to render the patient quite unrecognizable. The attacks would first begin by the violent twitching of the muscles of the face; her expression was horribly altered; the globes of the eyes were turned up under the eyelids, so as to leave only the sclerotics visible, and the angles of the mouth were retracted and fixed in a convulsive grin. The convulsive movement soon attacked the muscles of the body; after the face, the hands and arms, at first rigidly fixed, with the thumbs clenched into the palms; then the whole muscular system was thrown into rapidly recurring convulsive spasms. At 1:45 P. M. she had another, which lasted longer than the one prior. When in full headway, her radial arteries ceased to beat, while the action of the heart was very feeble, and the sounds, though normal, seemed distant, as though a pillow were placed between her chest and the ear of the auscultator. The involuntary muscles were implicated, as well as the voluntary, as was verified by the temporary arrest of respiration at the commencement of the attack, followed by irregular and hurried respiratory movements, producing a peculiar hissing sound. During the attacks sensibility was totally suspended, and the patient was unconscious even between the attacks, or in rather a stupefied condition.

On examination per vaginam, I found the os uteri dilated to a certain extent, yet the cervix was in reach. Seeing that the expulsion of the uterine contents alone could save the life of the suffering and completely exhausted primipara, I therefore resorted to instrumental delivery, and my only expedient was the high forceps operation. After placing the patient under complete anæsthesia with chloroform, then placing her near the edge of the bed, with the thighs flexed on the abdomen, an assistant at each knee, I proceeded. As stated above, the os was not fully dilated, but enough for the forceps to be scientifically introduced. I lubricated my left hand, then introduced it in the vagina as a guide, to avoid any possibility of injuring the cervix; then, with my hand in the vagina, introduced the lower blade of Hodge's lever forceps, previously oiling the outside of said blade. Finding the bladder distended, I introduced a catheter, and drew off about twenty ounces of urine (which proved, by testing, to be cloudy with albumen); this being done, I introduced the upper blade, which was done with more difficulty. Finally I succeeded in locking my instruments; then I began making traction downward toward the perineum. The pains being absent, my tractions were intermittent, so as to imitate nature as nearly as possible. When the caput succedaneum was pushing against the vulvæ, I elevated the forceps upward toward the mons veneris. The contents of the rectum and the little urine which still remained in the bladder made their escape through their normal orifices, which in all cases is exceedingly disagreeable to the attendants, especially the accoucheur; hence the necessity of keeping the bowels open during the last week of utero gestation and an enema the day before; but in this case time was too precious and life too dear to delay one moment for preparatory cleanliness. A fine boy (stillborn) was delivered at 4:35 P. M. When I first arrived, by auscultation the fœtal heart, also the placental soufle, could be distinctly heard. The umbilical cord was wound tightly around its neck; whether that produced its death, or whether it was seized with convulsions in utero remains a mystery, as either would have caused its death. Attempts were made to reanimate it by inflating the lungs, friction, etc., but without effect; the heart could not be made to beat even once. The cord was then severed.

She had only one convulsion after the delivery. Pulse 148, respiration normal, with occasional sighs; would notice no one; very restless and thirsty. Ten P. M., about the same; completely exhausted. October 21, two A. M., pulse 102; respiration 20; somewhat quiet; micturated during the night. Seven A. M., pulse 90; talks with attendants, knowing nothing about what happened, and asks why the doctor was there; sight good enough to distinguish faces. Uterus contracted nicely, and supporting bandage all right. Three P. M., some fever, which continued during the night. October 22; feeling better; no fever; pulse soft and slow. Relished a cup of coffee and some gruel; sight fully returned. Tongue sore from being bitten during the attacks; micturates without difficulty.

The precise pathology of eclampsia cannot be considered by any means satisfactorily settled. When, in the year 1843, Lever first showed that the urine in patients suffering from convulsions was generally highly charged with albumen, a fact which subsequent experience has amply confirmed, it was thought that a key to the etiology of the disease had been found. It was known that chronic forms of Bright's disease were frequently associated with retention of urinary elements in the blood, and not rarely accompanied by convulsions. The natural inference was drawn, that the convulsions of eclampsia were also due to toxæmia resulting from the retention of urea in the blood, just as in the uræmiæ of chronic Bright's disease; and this view was adopted and supported by the authority of Braun, Frerichs, and many other modern writers of eminence, and was pretty generally received as a satisfactory explanation of the facts. Frerichs modified it so far, that he held that the true toxic element was not urea, as such, but carbonate of ammonia, resulting from its decomposition; and experiments were made to prove that the injection of this substance into the veins of the lower animals produced convulsions of precisely the same character as eclampsia.

Dr. Hammond, of Maryland, subsequently made a series of counter experiments, which were held as proving that there was no reason to believe that urea ever did become decomposed in the blood in the way that Frerichs supposed, or that the symptoms of uræmia were ever produced in this way.

Spiegelberg has, more recently, again examined the question clinically, in a patient suffering from convulsions, in whose blood an excess of urea and ammonia was found, and by experiments on dogs, and maintains the accuracy of Frerich's views. Others have believed that the poisonous elements retained in the blood are not urea or the products of decomposition, but other extractive matters which have escaped detection. Broxton Hicks, who has recorded a considerable number of cases, says that albuminuria and convulsion are almost invariably combined, and must be explained in one of three ways:

First. That the convulsions are the cause of the nephritis.

Second. That the convulsions and the nephritis are produced by the same cause, *e. g.*, some detrimental ingredient circulating in the blood, irritating both the cerebro-spinal system and other organs at the same time.

Third. That the highly congested state of the venous system induced by the spasm of the glottis in eclampsia, is able to produce the kidney complication.

### TREATMENT.

On my arrival I gave inhalations of chloroform (Squibbs') at short intervals; seeing no decided effect, I ordered the following:

℞ Chloralis hydratis..................ʒv℈j.

Potass. bromidi. ..................℥j.

Tinct. opii deodorat...............ʒiv.

Aquæ fontis. ....................℥iijss.

M. Sig. Give a dessertspoonful in a tablespoonful of water every three hours.

This mixture acted like a charm in warding off the attacks. Fluid extract of ergot was given (drachm) in the second stage. After the expulsion of the placenta, which occurred twenty minutes after the delivery, a profuse hæmorrhage set up, which was checked by the administration of ergot, tamponing the vagina and kneading the uterus. It soon, however, assumed the size of a cricket ball; then the supporting bandage was properly adjusted. Ordered light but nourishing diet. Sulph. cinchonidia, grs. viij, every morning at six o'clock. October 21,

℞ Kal. iodidi ..................... ʒx.

Tr. belladonnæ ........ ... . ...ʒiv

Aquæ fontis........ ...........℥ijss.

M. Sig. Give a teaspoonful three times a day; also apply over each mamma a belladonna plaster 6 by 6, to produce galactischesis.

Ordered the vulva and external orifice of the vagina thoroughly lubricated, and the vagina syringed with tepid water, with a few minims of carbolic acid, and a warm towel to be kept over the parts.

October 22. Pulse normal; improving. Slight cephalalgia; complains of photophobia. 23d. Still improving; half ounce of oleum ricini; chloral mixture (formula above) to be given three times a day. 24th. Appetite returning; removed plasters from breasts. Sleeps well at night. Had two stools; slight fever in afternoon. 25th. Chloral mixture every morning. Slight increase of temperature; still complains of photophobia. 26th. Improving; appetite good; plasters removed; sat up awhile. 28th. Milk ceased oozing; no fever. Lochial discharges slight, pale greenish color and very sickening aroma; hence familiarly described as "green waters."

